# Novel biomarkers of ciliary extracellular vesicles interact with ciliopathy and Alzheimer’s associated proteins

**DOI:** 10.1080/19420889.2021.2017099

**Published:** 2021-12-25

**Authors:** Ashraf M. Mohieldin, Amal Alachkar, John Yates, Surya M. Nauli

**Affiliations:** a Department of Biomedical & Pharmaceutical Sciences, Chapman University, Irvine, CA 92618, USA; b Department of Medicine, University of California Irvine, Irvine, CA 92697, USA; c Department of Pharmaceutical Sciences, University of California Irvine, Irvine, CA 92697, USA; d Department of Molecular Medicine, The Scripps Research Institute, La Jolla, CA 92037, USA

**Keywords:** Ciliary extracellular vesicles, cytosolic extracellular vesicles, exosome, ectosome, primary cilia, ciliopathy disorders, Alzheimer disease, neurodegenerative disorders, bioinformatic, proteomics, biomarkers

## Abstract

Ciliary extracellular vesicles (ciEVs), released from primary cilia, contain functional proteins that play an important role in cilia structure and functions. We have recently shown that ciEVs and cytosolic extracellular vesicles (cyEVs) have unique and distinct biomarkers. While ciEV biomarkers have shown some interactions with known ciliary proteins, little is known about the interaction of ciEV proteins with proteins involved in ciliopathy and neurodegenerative disorders. Here, we reveal for the first time the protein-protein interaction (PPI) between the top five ciEVs biomarkers with ciliopathy and Alzheimer disease (AD) proteins. These results support the growing evidence of the critical physiological roles of cilia in neurodegenerative disorders.

## Introduction

Extracellular vesicles have been shown to exhibit numerous physiological functions. Ciliary extracellular vesicles (ciEVs) have been shown to play a key role in cardiovascular function in a murine model, resulting in hypotension, left ventricle hypertrophy, cardiac fibrosis, arrhythmia, and high mortality rate [1]. While the cellular-derived cytosolic EVs (cyEVs) have been widely studied, ciEV characteristics, including their size, proteins composition, and potential biomarkers, are not fully understood. Recently, we revealed for the first time the unique characteristics of ciEVs and cyEVs [2]. In addition to the different sizes between ciEVs and cyEVs, both vesicles exhibited unique biomarkers. However, the interaction of top-identified ciEVs with known human disorders is still not clear.

Ciliopathy and neurodegenerative disorders have long been associated with ciliary proteins. The mutation of specific ciliary genes (e.g., TMEM216, DCTN1, AHI1) leads to ciliopathy disorders and results in a wide range of phenotypes. These phenotypes may include neurological disorder features characterized by psychomotor disruptions and associated with dysmorphism (TMEM216 mutation), early-onset of Parkinson’s disease and depression (DCTN1 mutation), abnormal cerebellar development, and axonal decussation (AHI1 mutation) [3–5]. Alzheimer’s disease (AD) is one of the major neurodegenerative disorders characterized by dementia, impaired cognition, and language [6]. Significant protein interactions’ overlaps between cilia and AD have recently been revealed, suggesting an important role of cilia in AD [7]. It is believed that abnormality in cilia may result in the deteriorating of new memory formation in AD by affecting the dentate gyrus (DGy) neurogenesis [8–12]. However, the interaction between ciliopathy and AD with novel ciEV biomarkers has not been examined yet. We analyze here the novel ciEV biomarkers and their potential interaction with ciliopathy and AD disorders for the first time.

## Results and discussion

### Unique biomarkers of ciliary extracellular vesicles (ciEVs)

**Figure 1.**
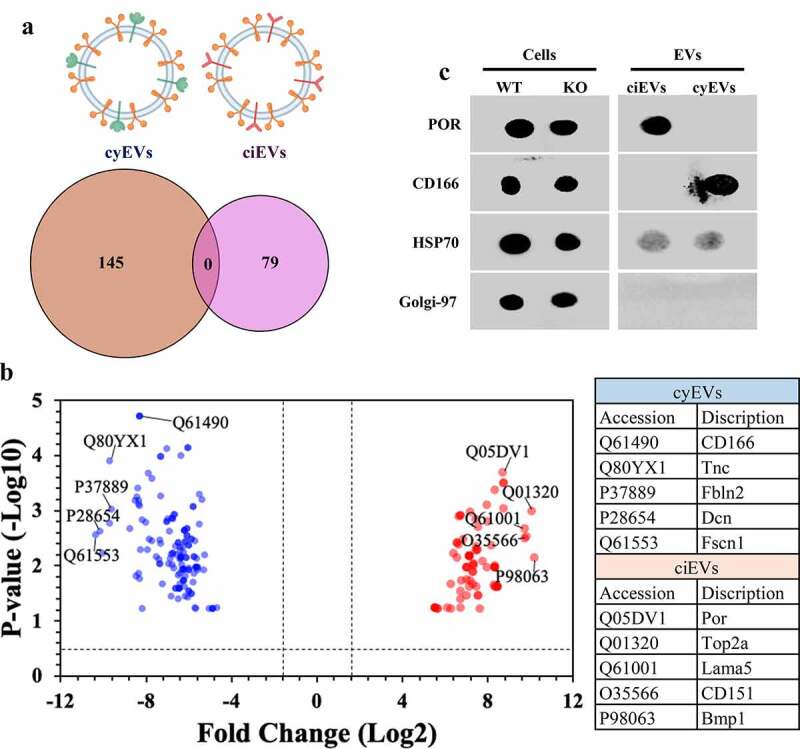
ciEVs and cyEVs have unique biomarkers

EVs isolated from ciliated (wild-type; ciEVs) and non-ciliated (*Ift88*; cyEVs) mouse endothelial cells were examined by proteomic analyses. The comparative proteomic analyses revealed 145 cyEV and 79 ciEV unique biomarkers (Figure 1a). To efficiently identify the top five biomarkers from both vesicles, we used the volcano plot to measure the comparative-proteome’s effect sizes and significance values. The annotated five data-points represented in the volcano plot had the largest distance (Manhattan) from the origin and above the thresholds indicated by the dashed lines (Figure 1b). To confirm the specificity of these biomarkers to each vesicle, we examined the expression of the top identified biomarkers in each vesicle, using immunoblot (dot blot) analyses (Figure 1c). The two selected biomarkers NADPH-cytochrome P450 reductase (POR) and CD166 antigen (CD166) demonstrated the exclusive expression in ciEV and cyEV lysates, respectively.

(a) EV isolation from ciliated (wild-type; ciEVs) and non-ciliated (*IFT88*; cyEVs) cells reveals unique biomarkers. (b) The volcano plot shows the top five distinctive identified biomarkers based on their pvalue and fold-change for cyEVs (blue color) and ciEVs (red color). The dot blot analyses show the expression of the top cyEV and ciEV biomarkers (CD166 and POR, respectively) in isolated EVs. HSP70 and Golgi-97 were used as positive and negative controls for extracellular vesicles, respectively.

### Interaction of novel ciEV biomarkers with associated human diseases

**Figure 2.**
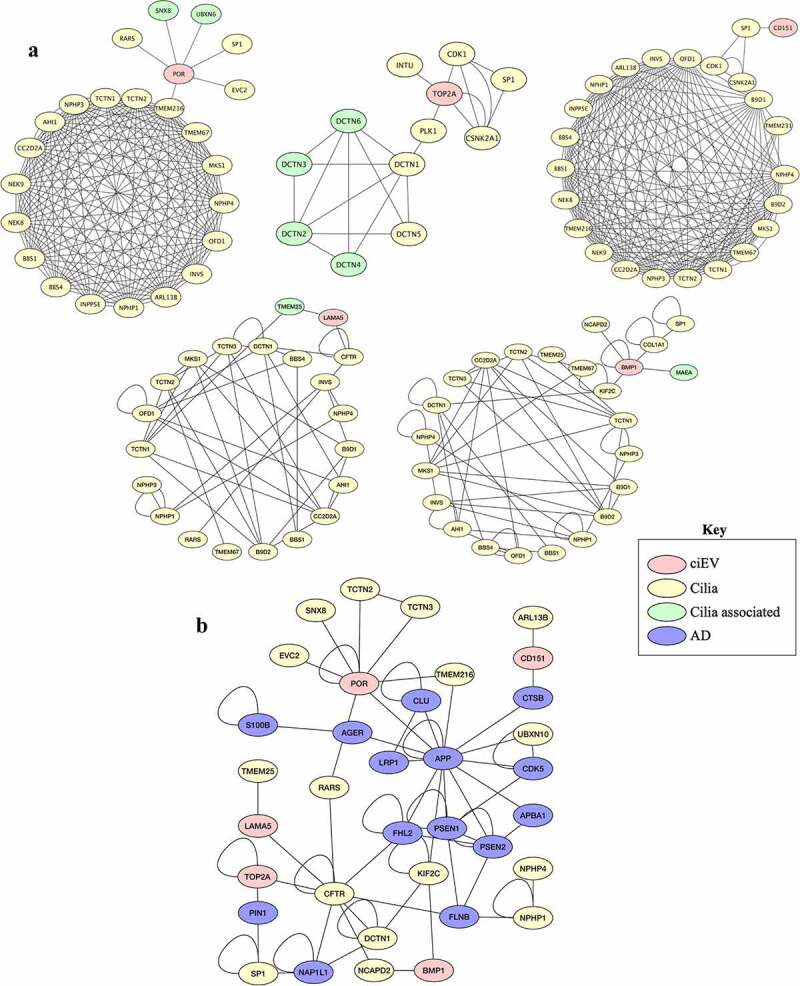
Bioinformatic analyses of ciliary extracellular vesicles (ciEVs) and potential interaction with AD biomarkers

We used the protein-protein interaction (PPI) network analyses to examine the potential interaction of the top ciEV biomarkers with known human diseases. Because ciliary proteins have long been associated with ciliopathy disorders, we first examined the interaction of top ciEV biomarkers with known ciliopathy proteins [1]. All top ciEV biomarkers (POR, TOP2A, LAMA5, CD151, BMP1) interacted with known ciliary or ciliary-associated proteins (Figure 2a). The ciliary-associated proteins here refer to a subfamily of known ciliary proteins that interact with ciliary protein. The result suggests that ciEV proteins could be involved in ciliopathy disorder. The potential direct or indirect interaction of ciEV biomarkers with known ciliopathy genes (e.g., TMEM216) further substantiated our previous findings that repression of ciEV genes (e.g., PGRMC2 and F11R) resulted in ciliopathic phenotypes [1].

(a) The PPI analyses show the top five ciEV biomarkers (light red color) interaction with known ciliary (light yellow color) and ciliary-associated (light green color) proteins.(b) The protein-protein network analyses show the top five ciEV biomarkers (light red color) interaction with known ciliary (light yellow color) and AD (light blue color) biomarkers.

Since cilia and EVs have been associated with Alzheimer disease (AD) [13–17], we again examined the interaction of top ciEV biomarkers with AD proteins (Figure 2b). Three of the top ciEV biomarkers (POR, TOP2A, CD151) have potential direct interactions with known AD markers (APP, PIN1, CTSB), respectively. The other two ciEV biomarkers (LAMA5 and BMP1) show potential indirect interactions through other ciliary proteins (CFTR, NCAPD2, KIF2C) with known AD biomarkers (FHL2, NAP1L1, FLNB, APP). Of note, the hedgehog signaling (Shh) regulated by primary cilia proteins (e.g., KIF3A) is thought to associate with AD [18–20]. The disruption of Shh signaling has been shown to induce the neurodegenerative disease, including AD by enhancing cognitive impairment and memory loss [21]. Interestingly, some of the ciEV and AD proteins presented in the PPI here (POR, TOP2a, CD151, LAMA5, CFTR, APP, PIN1, CTSB, FHL2) have been shown to associate with Shh signaling [15,21–30]. These novel findings support previous reports on the cross-talk between ciliary proteins and AD and the involvement in neurodegenerative mechanisms [7,10].

In summary, our follow-up analyses revealed for the first time the potential PPI of novel ciEV biomarkers with known ciliopathy and AD-associated proteins. However, cilia and EV proteins have been associated with other major neurodegenerative and psychiatric disorders, including Parkinson’s disease, schizophrenia, autism spectrum disorder, bipolar disorder, and major depressive disorder [14,31]. A comprehensive analysis is necessary to examine the causal relationship between ciEV genes and the neurodegenerative disorders. In conclusion, our findings substantiate the growing evidence that ciliary extracellular vesicles play a significant role in ciliopathy and neurodegenerative disorders.

### Methods

#### Immunoblot analyses

EVs were isolated from ciliated (ciEVs) and non-ciliated (*Ift88*; cyEVs) mouse knockout endothelial cells as previously described [2]. Briefly, cells were grown to reach 70–80% confluence. Next, cells were induced with a shear flow of 2.0 dyn/cm^2^ for 30 minutes. Growth media was then collected respectively and centrifuged at four different speeds: 300 × *g* for 10 minutes, 2,000 × *g* for 10 minutes 10,000 × *g* for 30 minutes, and 100,000 × *g* for 70 minutes. As the supernatants were collected in all first three rounds of centrifugation, the supernatants were discarded at the fourth round of centrifugation, and the vesicle pellets were re-suspended in radioimmunoprecipitation assay (RIPA) buffer. Next, the EV concentration of protein lysates were determined using Pierce BCA Protein assay kit, and proteins expressions were analyzed by dot blot approach.

Nitrocellulose membranes were blocked with 5% milk, incubated with primary antibodies (1:500, POR; 1:500, CD166; 1:500, golgi-97; and 1:100, HSP70) for overnight at 4°C and secondary antibodies (1:1000 for both anti-mouse and anti-rabbit) for 1 hour at room temperature, and imaged with the ChemiDoc XRS+ system.

#### Proteomic and bioinformatic

Upon a series of steps, isolated EVs from ciliated and non-ciliated endothelial cells were purified, resuspended in RIPA buffer, reduced, and digested for proteomic analyses. EVs protein samples were analyzed using liquid chromatography with tandem mass spectrometry (LC-MS-MS), as previously described [2]. The volcano plot was analyzed using the R project for statistical computing software (version 3.5.3). The PPI interaction network was analyzed with Cytoscape software (version 3.3.0). The network interaction was simplified to examine only interacted top novel ciEVs with ciliopathy and AD proteins.

## Figures and Tables

**Table:** 

	Acronym	Description
**ciEVMarkrs**	POR	NADPH-cytochrome P450 reductase
	TOP2A	Topoisomerase (DNA) II Alpha
	LAMA5	Laminin Subunit Alpha 5
	CD151	CD151 Antigen
	BMP1	Bone Morphogenetic Protein 1
**AD Markers**	APP	Amyloid beta (A4) precursor protein
	PIN1	Protein (peptidyl-prolyl cis/trans isomerase) NIMA interacting 1
	CTSB	Cathepsin B
	FHL2	Four and a half LIM domains 2
	NAP1L1	Nucleosome assembly protein 1-like 1
	FLNB	Filamin B, beta (actin binding protein 278)
**Cilia Markers**	CFTR	CF Transmembrane Conductance Regulator
	NCAPD2	Non-SMC Condensin I Complex Subunit D2
	KIF2C	Kinesin Family Member 2C
	TMEM216	Transmembrane protein 216
	DCTN1	Dynactin Subunit 1
	AHI1	Abelson Helper Integration Site 1
